# A call for better reporting of trials using surrogate primary endpoints

**DOI:** 10.1002/trc2.12340

**Published:** 2022-07-26

**Authors:** Anthony Muchai Manyara, Oriana Ciani, Rod S. Taylor

**Affiliations:** ^1^ MRC /CSO Social and Public Health Sciences Unit Institute of Health and Wellbeing University of Glasgow Glasgow UK; ^2^ SDA Bocconi School of Management Milan Italy; ^3^ MRC/CSO Social and Public Health Sciences Unit & Robertson Centre for Biostatistics, Institute of Health and Wellbeing University of Glasgow Glasgow UK

## 1 INTRODUCTION

Cummings et al. have recently reviewed current randomized controlled trials (RCTs) and drugs under development for Alzheimer's disease (AD) treatment.^1^ One of the key findings of this review was increased use of biomarkers as outcomes.^1^ Some of the biomarkers used, such as reduction in amyloid, are regarded as surrogate endpoints;^1^, ^2^ that is, substitutes and predictors of patient relevant outcomes^3^ such as death or disease progression. The cost of conducting trials to support development of treatments of chronic brain conditions is extremely high (US $42.5 billion in the past 25 years for AD) necessitating measures to lower trial cost.^4^ Additionally, development of such treatments is complex and difficult given it is dependent on demonstration of a health benefit on a highly progressive condition.^2^ Therefore, surrogate endpoints may improve trial efficiency and allow faster approval of treatments.

## 2 RISKS OF SURROGATES: THE ADUCANUMAB CONTROVERSY

Despite their benefits, using surrogate endpoints in trials and regulatory approval of interventions is controversial as they may not predict health benefits.^5^ A recent and highly publicized example is the approval of aducanumab for treatment of AD.^6^ Aducanumab was potentially the first government agency–approved AD treatment based on a surrogate endpoint (i.e., reduction in amyloid load).^7^ Conduct of two RCTs evaluating the treatment was stopped early due to lack of potential patient benefit but the US Food and Drug Administration (FDA) approved it based on effect on the surrogate endpoint in one of the trials leading to public criticism and resignation of three members of the FDA approval committee.^6^ Such positive effects on surrogate endpoints but failure to predict health benefits could be due to the patient‐relevant final outcome being affected by disease causal pathways that are not mediated by the surrogates.^5^ Interventions approved based on surrogates rather than patient‐relevant final outcomes may not be cost effective and may lead to controversy in payer/reimbursement decisions. Indeed, despite FDA approval of aducanumab, Medicare (the federal health plan for older Americans) resolved to only pay for the treatment for patients enrolled in trials.^8^ Therefore, RCTs using a primary surrogate endpoint should be more transparent in their reporting, that is, clear statement of using a surrogate primary endpoint, validity, and limitations of surrogate used.^9^ However, the report of the two RCTs that evaluated aducanumab^10^ had no mention of “surrogate” and although they presented a rationale of using amyloid load, it is controversial and unprecedented to consider amyloid load as a valid surrogate in AD trials.^7^ Such inadequate reporting in RCTs that use surrogate endpoints has been previously reported: a review of 626 trials published in 2005 and 2006 found that 109 (17%) used a surrogate primary endpoint and of these, only 38 (35%) discussed the validity of the surrogate endpoint.^9^

## 3 NEED FOR IMPROVED REPORTING

Implementing reporting guidelines can improve the completeness of trial reporting. Two widely used guidelines are the SPIRIT (Standard Protocol Items: Recommendations for Interventional Trials) 2013 statement: a 33‐item checklist used for reporting RCT protocols (www.spirit‐statement.org) and CONSORT (Consolidated Standards of Reporting Trials) 2010 Statement: a 25‐item checklist used to improve reporting of conducted trials (www.consort‐statement.org). However, these guidelines and their relevant extensions do not provide guidance for the reporting of surrogate primary endpoints.

**FIGURE 1:**
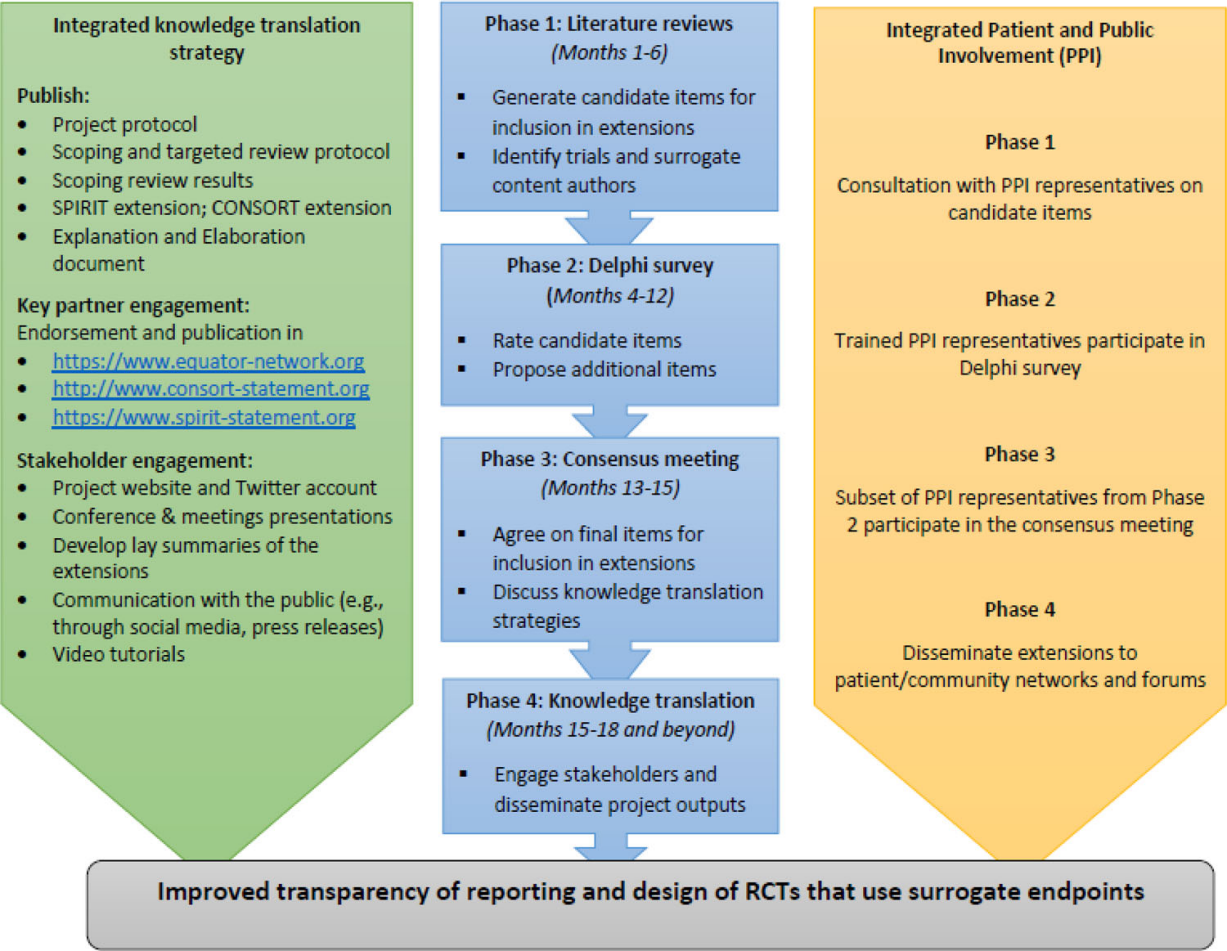
Project phases, timelines, activities in each phase (middle), with integrated knowledge translation (left) and patient and public involvement (right). Timelines include preparatory work before start of each phase. CONSORT, Consolidated Standards of Reporting Trials; RCT, randomized controlled trial; SPIRIT, Standard Protocol Items: Recommendations for Interventional Trials

Therefore, we announce a project that commenced in January 2022 to develop SPIRIT and CONSORT extensions specific to surrogate endpoints (“SPIRIT‐SURROGATE” and “CONSORT‐SURROGATE”). These extensions will improve the reporting of RCT protocols and reports that use a surrogate primary endpoint and allow for better scrutiny of surrogacy evidence. Figure 1 summarizes the project phases and timelines.

To make the development inclusive and developed extensions as usable as possible, we would like to invite various stakeholders (trial methodologists, journal editors, the health‐care industry, regulators and payers, and patient/public representative groups), particularly those with interest or experience in using surrogate endpoints in trials, to contribute. Readers can follow project progress and indicate their interest in participation through our project webpage (https://www.gla.ac.uk/spirit‐consort‐surrogate).

## CONFLICTS OF INTEREST

The authors declare no conflicts.
